# The role of GLP-1 receptor agonists in IBD-related surgery and IBD-related complications of inflammatory bowel disease among patients with metabolic comorbidities: a systematic review and meta-analysis

**DOI:** 10.3389/fmed.2025.1621958

**Published:** 2025-08-21

**Authors:** Mengqi Yang, Yujia Huo, Zhining Liu, Guannan Bai, Dongjuan He, Lin Zhang

**Affiliations:** 1 The Faculty of Pharmacy and Pharmaceutical Science, Monash University, Melbourne, VIC, Australia; 2 The School of Public Health and Preventive Medicine, Monash University, Melbourne, VIC, Australia; 3 Suzhou Industrial Park Monash Research Institute of Science and Technology, Monash University, Suzhou, China; 4 Monash University-Southeast University Joint Research Institute (Suzhou), Southeast University, Suzhou, China; 5 Department of General Surgery, The Second Affiliated Hospital of Anhui Medical University, Hefei, China; 6 Department of Child Health Care, Children’s Hospital, National Clinical Research Center for Child Health, Zhejiang University School of Medicine, Hangzhou, China; 7 Department of Endocrinology, The Second People's Hospital of Quzhou, Quzhou, China

**Keywords:** inflammatory bowel disease, IBD, Crohn’s disease, ulcerative colitis, GLP-1RA, cohort studies, systematic review, meta-analysis

## Abstract

**Background:**

Glucagon-like peptide-1 receptor agonists (GLP-1RAs) are widely used for type 2 diabetes and obesity, and emerging evidence suggests potential immunomodulatory effects. However, few studies have evaluated their role in inflammatory bowel disease (IBD), and no comprehensive clinical trials exist. This meta-analysis aimed to assess the association between GLP-1RA use and IBD-related surgeries and complications.

**Methods:**

A systematic literature search was conducted in PubMed, Embase, and Web of Science from inception to March 2025. Cohort studies comparing IBD patients treated with GLP-1RAs versus non-users were included. Two reviewers independently performed study selection and data extraction. Random-effects meta-analyses were performed using log-transformed effect sizes. Heterogeneity was assessed using I^2^ and τ^2^. Publication bias was evaluated through funnel plots, and certainty of evidence was graded using the GRADE framework.

**Results:**

Six studies were included, providing eight effect estimates (two studies contributed two cohorts each). Three of the six included studies (50%) were non–peer-reviewed conference abstracts, which may affect interpretability. For IBD-related surgery (4 effect estimates), GLP-1RA use was significantly associated with lower risk (pooled estimate: 0.45; 95% CI: 0.35–0.59; I^2^ = 38.1%). For IBD-related complications (4 estimates), GLP-1RA use showed a non-significant trend toward benefit (estimate: 0.39; 95% CI: 0.15–1.03), with high heterogeneity (I^2^ = 98.9%). Sensitivity analysis supported robustness for surgery but revealed instability in complication outcomes. Funnel plots showed no publication bias for surgery, but asymmetry was noted for complications.

**Conclusion:**

GLP-1RA use may reduce the risk of IBD-related surgery among IBD patients with metabolic comorbidities. Findings for IBD-related complications should be interpreted with caution due to substantial heterogeneity and the inclusion of abstract-only studies. Further prospective research is warranted.

**Systematic review registration:**

This systematic review was prospectively registered with the International Prospective Register of Systematic Reviews (PROSPERO), under registration ID CRD420251015882. The full protocol is publicly accessible through the PROSPERO database at https://www.crd.york.ac.uk/prospero/. No amendments were made to the original protocol. Any changes arising during the peer-review process will be transparently documented in the final publication.

## 1 Introduction

Inflammatory bowel disease (IBD), comprising Crohn’s disease (CD) and ulcerative colitis (UC), is a chronic immune-mediated condition characterized by recurrent gastrointestinal inflammation and progressive mucosal damage (1–3). UC typically presents as continuous superficial inflammation confined to the colonic mucosa, while CD features transmural inflammation that may involve any part of the gastrointestinal tract, often resulting in complications such as strictures, fistulas, or abscesses (2, 3). Despite advances in corticosteroids, immunosuppressants, and biologics targeting tumor necrosis factor (TNF) or interleukins, treatment outcomes remain suboptimal. This may reflect disease heterogeneity, unpredictable responses, immunogenicity, and cumulative long-term toxicities (4, 5). Moreover, current therapies are largely focused on suppressing inflammation rather than modifying the underlying disease course. Consequently, there is an urgent need to explore novel treatment strategies that offer durable efficacy, mucosal healing, and a more favourable safety profile.

In recent years, glucagon-like peptide-1 receptor agonists (GLP-1 RAs), originally developed for the treatment of type 2 diabetes and obesity, have drawn increasing attention in the context of chronic inflammatory diseases (6, 7). Beyond their well-established metabolic benefits, GLP-1 RAs have demonstrated immunomodulatory properties across multiple preclinical and clinical settings (6, 8). Mechanistically, GLP-1 signaling has been shown to inhibit key pro-inflammatory pathways, particularly by suppressing muclear factor-κB (NF-κB) activation and downregulating cytokines such as TNF-α, IL-6, IL-1β, IL-12, and IL-22 (9–11). In addition, GLP-1 may exert protective effects on intestinal homeostasis by enhancing epithelial barrier integrity, promoting mucosal healing, and modulating the gut microbiota—features that are directly relevant to the pathogenesis of IBD (6, 9).

A growing body of animal evidence supports these effects (9, 10, 12). In dextran sulfate sodium (DSS)-induced murine colitis models, both liraglutide and semaglutide significantly attenuated disease severity, reduced histopathological inflammation, and suppressed colonic cytokine expression (9). More recently, the application of GLP-1-loaded nanocarriers has been shown to enhance local drug delivery to inflamed intestinal tissue, thereby improving anti-inflammatory efficacy while minimizing systemic exposure and off-target effects (12).

Preliminary human data are also promising. Retrospective cohort studies and real-world analyses reported GLP-1 RA use has been associated with lower rates of IBD-related hospitalizations, surgeries, and corticosteroid prescriptions in patients with type 2 diabetes or obesity (13–15). Several large-scale observational studies have further highlighted potential reductions in the need for intestinal resections and IBD disease-related complications among GLP-1 users, particularly in ulcerative colitis patients with elevated body mass index or metabolic comorbidities (7, 15–19). Improvements in inflammatory markers such as C-reactive protein (CRP) and fecal calprotectin have also been observed (14, 15, 18). However, these findings are based on a limited number of non-randomized studies. Most available data derive from single-center or non-replicated cohort analyses, with limited validation across diverse populations or study designs. Therefore, the clinical implications of GLP-1 RA use in IBD remain uncertain and warrant systematic evaluation.

To address this gap, we conducted a systematic review and meta-analysis to critically evaluate whether GLP-1 receptor agonists are associated with improved clinical outcomes in patients particularly those with metabolic comorbidities, with a specific focus on two key endpoints: IBD-related surgery and IBD-related complications. Given the absence of randomized controlled trials in this area, a comprehensive synthesis of cohort-based and real-world evidence is critical to inform clinical decision-making and guide future interventional studies.

## 2 Methods

This systematic review was prospectively registered in the International Prospective Register of Systematic Reviews (PROSPERO; registration number CRD420251015882) and conducted in accordance with the PRISMA 2020 guidelines (20, 21).

### 2.1 Eligibility criteria

Studies were eligible if they met the following criteria:

- Population: Adults with a confirmed diagnosis of inflammatory bowel disease (IBD), including ulcerative colitis (UC) or Crohn’s disease (CD), with or without type 2 diabetes mellitus (T2D) or obesity.
- Exposure: Use of any glucagon-like peptide-1 receptor agonist (GLP-1 RA), such as liraglutide or semaglutide.
- Comparison: IBD patients not receiving GLP-1 RAs.
- Outcomes: IBD-related complications (e.g., hospitalisation, corticosteroid use, intestinal obstruction) and/or IBD-related surgery.
- Study design: Observational cohort studies.

We excluded randomized controlled trials, case reports, reviews, and animal studies. Conference abstracts were included only if they explicitly described a cohort study design, provided sufficient methodological detail (e.g., data source, outcome definitions), and reported extractable outcome estimates. Only studies published between January 2005 and March 2025 were considered for inclusion.

### 2.2 Search strategy

A comprehensive literature search was conducted in PubMed, Embase, and Web of Science to identify relevant studies published from January 2005 to March 15, 2025. The year 2005 was chosen as the start date because it marks the period when GLP-1 receptor agonists were first approved and introduced into clinical practice, allowing us to capture studies reflective of real-world use and clinical relevance. The search strategy included both Medical Subject Headings (MeSH) and free-text keywords covering glucagon-like peptide-1 receptor agonists (e.g., “GLP-1 RA,” “semaglutide,” “liraglutide”), inflammatory bowel disease (e.g., “ulcerative colitis,” “Crohn’s disease”), and study design descriptors (e.g., “real-world evidence,” “observational study,” “cohort study”). Terms referring to real-world, clinical trials and cohort studies were combined using the Boolean operator ‘OR’, and then logically connected to IBD- and GLP-1-related terms using ‘AND’ to ensure specificity to non-randomised, real-world evidence.

The search was restricted to human studies published in English. The complete search strategies for each database are provided in Supplementary Table 1. Although the search strategy included terms related to both randomized and observational studies to ensure comprehensive retrieval, no eligible randomized controlled trials were identified during screening. As such, the final synthesis was based exclusively on cohort-based observational studies.

All retrieved records were imported into EndNote for deduplication. Two reviewers independently screened titles and abstracts, followed by full-text reviews of potentially relevant articles. Disagreements during the screening process were resolved by discussion, and unresolved cases were adjudicated by a third reviewer.

### 2.3 Data extraction and quality assessment

Data extraction was performed independently by two reviewers using a standardized Microsoft Excel spreadsheet. The primary outcomes of interest were IBD-related surgeries and complications. “Complications” were defined based on author-reported composite outcomes explicitly labeled as “IBD-related complications,” typically encompassing hospitalizations, corticosteroid use, or intestinal obstruction. When results were presented at multiple time points or using different statistical models, we prioritized the most fully adjusted effect estimate at the longest follow-up duration. No automation tools were used in the data extraction process, and no attempts were made to contact study authors for additional data.

In addition to outcome data, we extracted relevant study characteristics such as article title, publication year, journal name and impact factor, study design, sample size (if reported), effect estimates (e.g., risk ratios [RR], odds ratios [OR], hazard ratios [HR]) with 95% confidence intervals (CI), authors’ key conclusions, proposed mechanisms by which GLP-1 RAs may influence IBD, and any evidence of symptom improvement. Comparator groups were extracted as defined by each study. All studies used IBD patients not receiving GLP-1RA therapy as the primary reference group, and none used active comparators such as other glucose-lowering medications.

Risk of bias was assessed for each included cohort study using the Newcastle-Ottawa Scale (NOS), which evaluates methodological quality across three domains: selection of participants, comparability of cohorts, and ascertainment of outcomes (22). Each study was scored from 0 to 9, and those scoring below 7 were considered lower quality. Supplementary Table 1 provides the detailed scoring all NOS domains (selection, comparability, and outcome) for each included study. Quality assessments were independently conducted by two reviewers, with discrepancies resolved through discussion with a third reviewer.

The certainty of evidence for each primary outcome was assessed using the Grading of Recommendations Assessment, Development and Evaluation (GRADE) approach (23). This included evaluation of five domains: risk of bias, inconsistency, indirectness, imprecision, and publication bias. GRADE ratings were assigned as high, moderate, low, or very low certainty. Full GRADE profiles are provided in Supplementary Table 2.

### 2.4 Statistical analysis

Studies were eligible for inclusion in the meta-analysis if they reported effect estimates (RR, OR, or HR) with 95% confidence intervals for either IBD-related surgery or complications. Prior to pooling, all effect estimates were log-transformed to stabilize variance and ensure comparability across studies.

When effect sizes were reported for non-GLP-1 RA users, we calculated inverse values to reflect the direction of association favoring GLP-1 RA users. For one study that did not report a 95% CI, we derived the interval from available raw data, including event counts and sample size. No other imputation was performed. A random-effects model was chosen to account for anticipated clinical and methodological heterogeneity across studies. Considering heterogeneity for the surgical outcome was moderate, we retained this model for consistency and to accommodate differences in populations, data sources, and analytic approaches (24). Forest plots were generated to present both individual study estimates and pooled effect sizes. Between-study heterogeneity was assessed using the I^2^ statistic. Leave-one-out sensitivity analyses were performed by iteratively excluding one study at a time and recalculating the pooled estimate. This was used to evaluate the influence of each individual study on the meta-analytic results. Results were presented graphically as influence plots (Supplementary Figure 1).

To explore potential sources of heterogeneity, we conducted meta-regression analyses using multiple moderators simultaneously in a single model. The included moderators were: (i) underlying condition (type 2 diabetes, obesity, or both), (ii) data source (e.g., claims vs. electronic medical records), and (iii) IBD subtype (ulcerative colitis vs. Crohn’s disease). This multivariable approach allowed assessment of their independent contributions to between-study heterogeneity. Although the number of studies per outcome was below the threshold typically recommended for formal publication bias testing, we generated funnel plots and conducted Egger’s regression as exploratory analyses to qualitatively assess small-study effects (25). Potential reporting bias was qualitatively considered during evidence appraisal. All statistical analyses were conducted using Stata version 18.0 (StataCorp, College Station, TX, USA). Prior to meta-analysis, key study information, summary tables, and extracted outcomes were organized and processed in Microsoft Excel. Statistical significance was defined as a 95% confidence interval not crossing the null value (RR or OR = 1.0). Clinical significance thresholds were not pre-specified; effect estimates were interpreted in the context of consistency, magnitude, and potential relevance to IBD-related outcomes.

## 3 Results

### 3.1 Study selection

A total of 161 records were identified from electronic databases (17 from PubMed, 69 from Embase, and 75 from Web of Science). After removing duplicates, 146 unique records remained. Following title and abstract screening, 38 studies underwent full-text assessment, and 22 met all inclusion criteria and were included in the review. Of the 22 studies eligible for systematic review, 6 were included in the meta-analysis based on availability of extractable effect estimates for predefined primary outcomes. The remaining 16 studies were excluded from synthesis due to the absence of effect sizes or outcome mismatch, as detailed in Supplementary Table 4.

**Figure 1:**
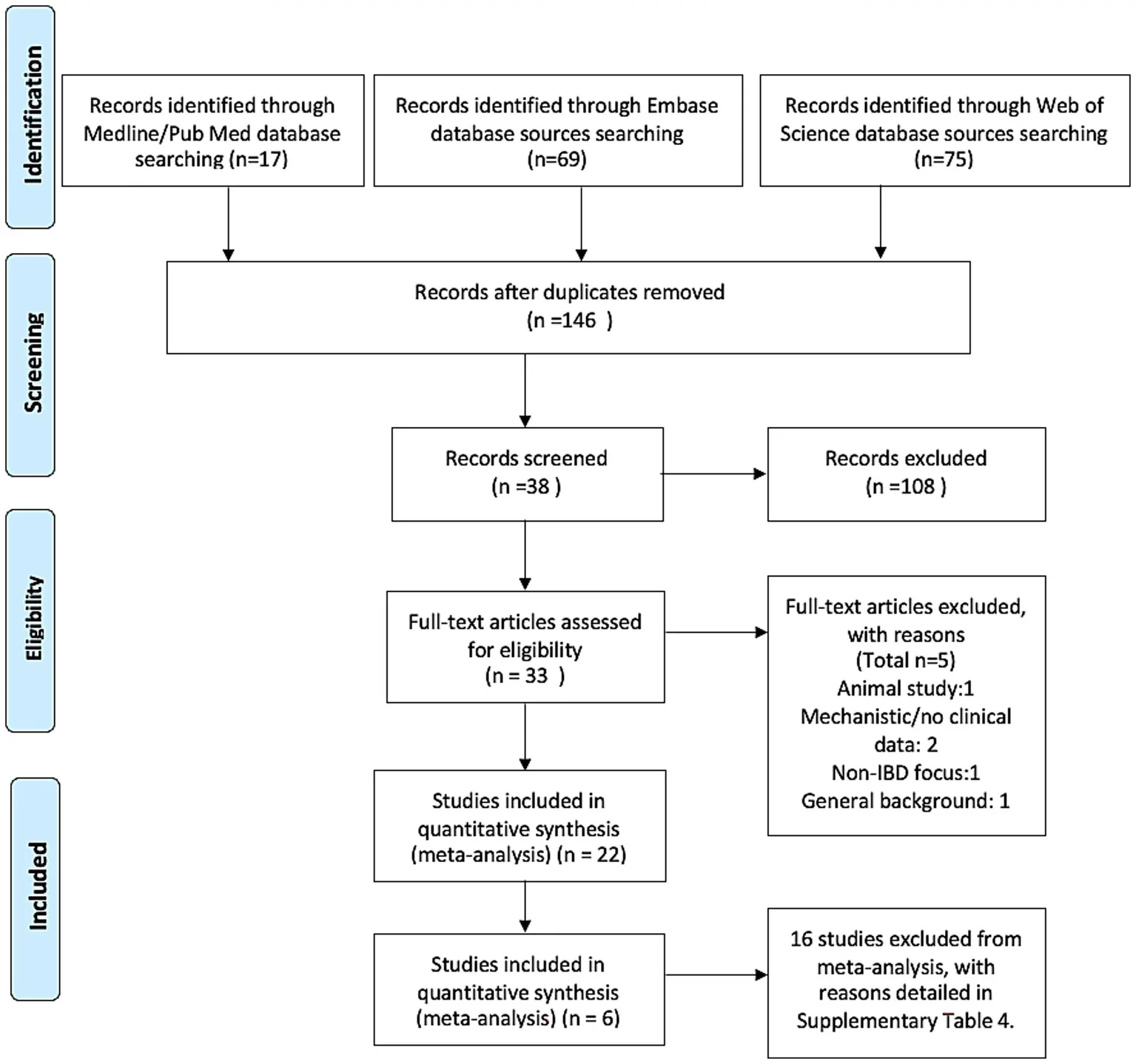
PRISMA flow diagram.

Six studies were eligible for meta-analysis. Four studies contributed data on IBD-related surgeries, and three on IBD-related complications, with one study providing data for both outcomes. The study selection process is summarized in Figure 1 (PRISMA flow diagram), with further detail on exclusion from synthesis provided in Supplementary Table 4. Demographic data such as mean age and sex distribution were inconsistently reported across studies. Of the six included cohorts, only two provided usable information: Desai2024 reported a mean age of 48.9 ± 12.8 years and 76.1% female participants; Villumsen2021 reported that 71.4% of patients were aged 30–65, with 45.3% female. The remaining studies, primarily published as conference abstracts, did not report demographic characteristics in sufficient detail. As a result, these variables were not included in Table 1 but are acknowledged as a limitation in assessing generalizability. In addition, although three of the included studies were published as conference abstracts, all explicitly described a retrospective cohort design based on real-world data from the TriNetX platform. These studies met our pre-specified inclusion criteria due to the availability of outcome estimates and the presence of sufficient methodological detail to assess risk of bias. Their inclusion was further justified by the limited number of full-text studies available on this topic and the critical value they contributed to maintaining the minimal threshold (n ≥ 3) for meta-analysis. Nevertheless, their abstract-only publication status is acknowledged as a limitation.

**Table 1:** Characteristics of studies included in the meta-analysis.

Study ID	Study design	Database	IBD type	Population type	Sample size after PSM	Outcome reported
Villumsen et al (7)	Population-based Cohort	Danish National Registries	Mixed	T2D	3,751	IBD-related complication
Adekolu et al. (17)	Population-based Cohort	TriNetX	Mixed	Obesity	14,840	IBD-related complication
Nieto et al. (16)	Retrospective Cohort	TriNetX	UC	Obesity	4,408	IBD-related complication
Abboud et al. (18)	Retrospective Cohort	TriNetX	Mixed	General IBD	5,508	IBD-related surgery & complication
Saadeh et al. (15)	Retrospective Cohort	TriNetX	Mixed	T2D	15,778	IBD-related surgery
Desai et al. (14, 19)	Retrospective Cohort	TriNetX	UC, CD	Obesity	2,270	IBD-related surgery (2 arms)

This table summarizes the key features of the six studies included in the meta-analysis, including study design, population, IBD subtype, data source, and outcomes assessed.

### 3.2 Study characteristics

The six studies included in the meta-analysis were all retrospective cohort studies based on real-world data sources, including large-scale registries such as TriNetX and national databases. The study populations were heterogeneous: some included IBD patients with obesity, others with type 2 diabetes, and one included general IBD patients regardless of metabolic comorbidity.

Outcomes assessed varied, with some studies focusing on surgical events (e.g., colectomy, intestinal resection) and others on complications (e.g., hospitalization, steroid dependence, or perforation). Study characteristics are summarized in Table 1.

### 3.3 Risk of Bias

All included studies were assessed using the Newcastle-Ottawa Scale (NOS) (22). Total scores ranged from 7 to 9 out of a maximum of 9. Two studies were rated as high quality, three as moderate to high, and one as moderate. Most studies scored well in the selection domain, while comparability was limited in some cases due to incomplete covariate adjustment. A detailed breakdown of domain-level scores is provided in Supplementary Table 2.

### 3.4 Individual study results

Effect estimates from the six studies are presented in log-transformed form to facilitate meta-analysis. Across studies, GLP-1RA use was consistently associated with a reduction in IBD-related surgeries and complications. Log(OR) values ranged from −0.21 to −2.16, and most 95% confidence intervals excluded the null value (see Table 2).

**Table 2:** Effect estimates for IBD-related surgeries and complications.

Study ID	Outcome	Effect estimate (log OR)	95% CI (Lower–Upper)
Villumsen et al (7)	IBD-related Complication	−0.65	−0.87 to −0.43
Adekolu et al. (17)	IBD-related Complication	−2.16	−2.34 to −1.98
Nieto et al. (16)	IBD-related Complication	−0.76	−1.08 to −0.42
Abboud et al. (18)	IBD-related Complication	−0.21	−0.36 to −0.07
Abboud et al. (18)	IBD-related Surgery	−0.63	−0.94 to −0.33
Saadeh et al. (15)	IBD-related Surgery	−1.08	−1.41 to −0.75
Desai et al. (14)	IBD-related Surgery	−0.99	−1.97 to −0.03
Desai et al. (19)	IBD-related Surgery	−0.60	−1.02 to −0.17

Summary of log-transformed odds ratios (log OR) and corresponding 95% confidence intervals (CI) for each included study.

### 3.5 Meta-analysis results

#### 3.5.1 IBD-related surgery

**Figure 2:**
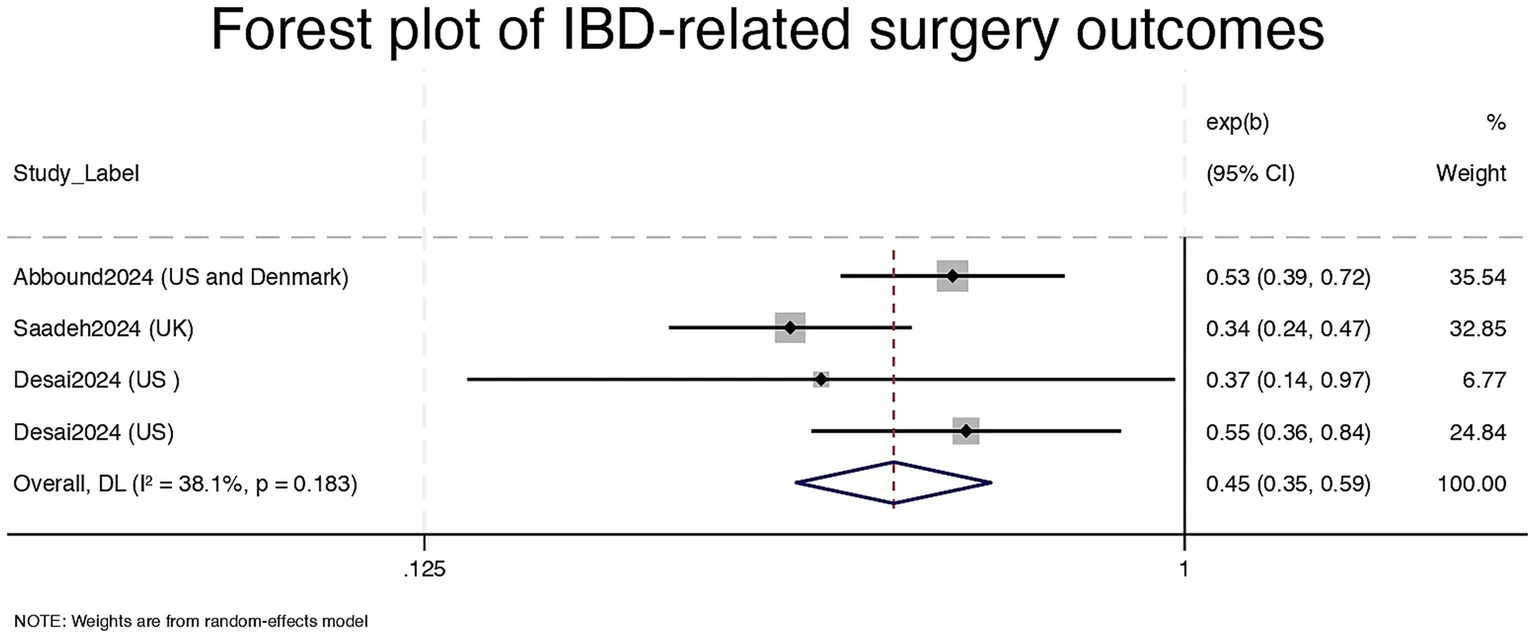
Forest plot of IBD-related surgery outcomes. CI = Confidence Interval; exp (b) = exponentiated regression coefficient; DL = DerSimonian and Laird random-effects model.

Four studies contributed data on IBD-related surgical outcomes (7, 15, 18, 19). The pooled effect estimate showed that GLP-1RA use was associated with a 55% reduction in the risk of IBD-related surgery (RR = 0.45, 95% CI: 0.35–0.59), with a log-transformed effect size of −0.80 (95% CI: −1.06 to −0.53, *p* < 0.001). Heterogeneity was moderate (I^2^ = 38.1%, τ^2^ = 0.0272), and Cochran’s Q was non-significant (*p* = 0.183), indicating consistent findings across studies. All included studies reported a protective effect (exp(b) < 1) (see Figure 2). A fixed-effect meta-analysis was also conducted to assess the robustness of findings. The pooled odds ratio under the fixed-effect model was 0.47 (95% CI: 0.39–0.57), which was consistent with the random-effects estimate. This supports the reliability of the observed protective association between GLP-1RA use and IBD-related surgery (see Supplementary Figure 2)

#### 3.5.2 IBD-related complications

**Figure 3:**
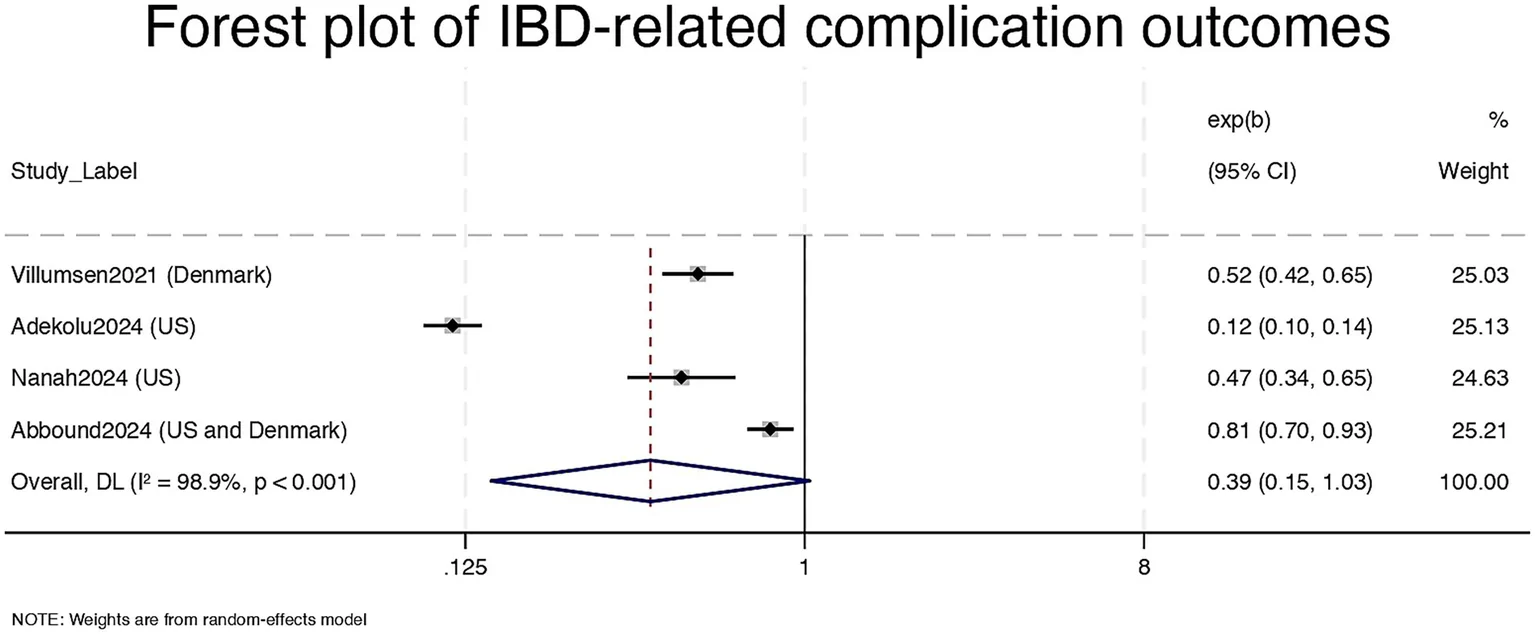
Forest plot of IBD-related complication outcomes. CI = Confidence Interval; exp (b) = exponentiated regression coefficient; DL = DerSimonian and Laird random-effects model.

Three studies reported on IBD-related complications such as hospitalization, perforation, or corticosteroid use, Desai2024 provide data for UC and CD separately (16–18). The pooled estimate was 0.39 (95% CI: 0.15–1.03), log(ES) = −0.94, with a *p*-value of 0.058, indicating a non-significant but potentially meaningful risk reduction. However, heterogeneity was extremely high (I^2^ = 98.9%, τ^2^ = 0.9769; Q = 284.69, *p* < 0.001). Effect estimates ranged widely, with one study showing a very strong association (RR = 0.12) and another closer to the null (RR = 0.81) (see Figure 3). We did not conduct a fixed-effect model for the complication outcome due to extreme heterogeneity (I^2^ = 98.9%), which violates the assumptions of such a model and could produce misleading results. All model-based sensitivity analyses for this outcome were thus restricted to the random-effects framework.

### 3.6 Meta-regression

To explore potential sources of heterogeneity in the complication outcome, we conducted a meta-regression using multiple moderators simultaneously (26). The covariates included underlying condition (T2D, obesity, or general IBD), data source (TriNetX vs. national registry), and IBD subtype (UC, CD, or mixed). None of these variables significantly explained the between-study variance (all *p* > 0.2), and the adjusted R^2^ was low, suggesting unmeasured factors may underlie the observed heterogeneity. For the IBD-related surgery outcome, sensitivity analysis showed highly consistent results across studies. In contrast, for the IBD-related complication outcome, exclusion of individual studies led to noticeable shifts in the pooled effect size and CI width, indicating that findings were driven by one or more studies with extreme estimates.

### 3.7 Sensitivity analyses

For the surgery outcome, leave-one-out analysis demonstrated robust results: removing any single study did not substantially change the pooled estimate or confidence interval. All estimates remained statistically significant and consistently favored GLP-1RA use (see Supplementary Figure 3). Desai2024 appears twice due to distinct cohorts or endpoints reported within the same publication.

In contrast, the complication outcome was more sensitive (see Supplementary Figure 4). Exclusion of Abboud2024 notably widened the confidence interval and attenuated the point estimate, suggesting that results for this outcome were more vulnerable to small-study effects or methodological variability.

Meta-regression analyses using multiple moderators did not reveal any significant contributors to heterogeneity (26). All confidence intervals crossed the null, and adjusted R^2^ was low.

### 3.8 Reporting Bias

**Figure 4:**
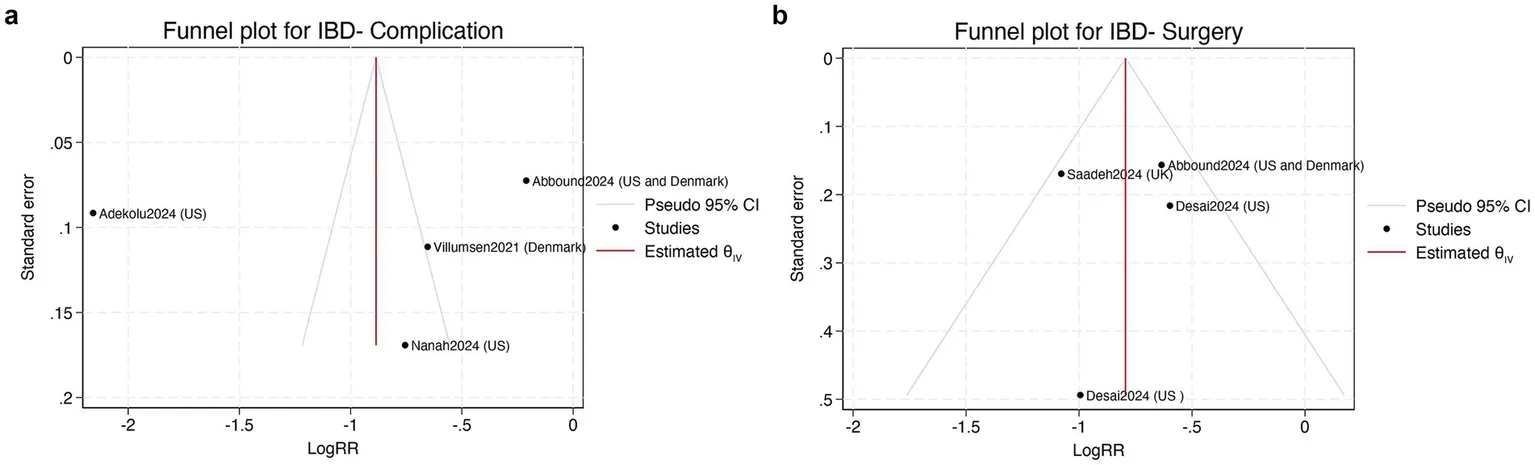
**(a)** Funnel plot assessing publication bias of IBD-related surgery (left); **(b)** Funnel plot assessing publication bias of IBD-related complications (right). LogRR = log risk ratio; CI = confidence interval; θ_IV_ = estimated pooled effect; SE = standard error.

Publication bias was evaluated through funnel plot inspection and Egger’s regression (25). For the surgery outcome in Figure 4a, the funnel plot appeared symmetrical, and Egger’s test was not significant (*p* > 0.1), suggesting a low likelihood of publication bias. For complications, however, the funnel plot showed clear asymmetry, with one outlying study reporting an extreme effect size. While this may reflect small-study effects or selective reporting, the small number of studies (n < 10) limits interpretability (see Figure 4b).

### 3.9 Certainty of evidence

Using the GRADE framework, the certainty of evidence was evaluated at the outcome level for both endpoints. For IBD-related surgery, the certainty was rated as low. Although the evidence was derived from observational studies, the results were consistent across studies, remained robust in sensitivity analyses, and demonstrated only moderate heterogeneity. In contrast, for IBD-related complications, the certainty of evidence was rated as low, primarily due to very high heterogeneity, instability of the pooled estimate in sensitivity analyses, and potential risk of reporting bias. Additional details are provided in Supplementary Table 3.

## 4 Discussion

This systematic review and meta-analysis evaluated the potential impact of GLP-1RAs on clinical outcomes in patients with IBD. Based on four retrospective cohort studies, GLP-1RA use was statistically significantly associated with lower risk in IBD-related surgery risk (pooled RR = 0.45, 95% CI: 0.35–0.59) and a potential, though statistically non-significant, decrease in complications (RR = 0.39, 95% CI: 0.15–1.03) (7, 15, 18, 19). The surgical outcome showed consistent directionality, low heterogeneity (I^2^ = 38.1%), and robust sensitivity to study exclusion, indicating high reliability and potential clinical relevance. Minor differences in comparator definitions across studies, such as baseline comorbidities or treatment history, may have contributed to residual heterogeneity, particularly for the complication outcome. These findings suggest that GLP-1RAs may confer immunomodulatory benefits beyond their metabolic effects.

Preclinical findings support this association. In a DSS-induced murine colitis model, liraglutide treatment significantly reduced histological inflammation scores (from 7.4 ± 0.3 to 2.2 ± 0.5, *p* < 0.001), and suppressed TNF-α and IL-6 expression by over 60% (9). *In vitro* evidence further suggests that GLP-1RAs enhance mucosal integrity by upregulating tight junction proteins such as claudin-1 and occludin in intestinal epithelial cells (27). Additionally, a recent study using a DSS-colitis mouse model demonstrated that nanoparticle-based delivery of anti-inflammatory agents (e.g., PLGA-encapsulated tofacitinib) enhanced drug accumulation in the inflamed colon and improved colitis severity compared to free drug administration (28). These mechanistic insights support the hypothesis that GLP-1RAs, either directly or via optimized delivery systems, may contribute to preservation of mucosal damage and lower surgical risk in IBD. However, as most of these mechanisms have been demonstrated in preclinical models, caution is warranted when extrapolating their relevance to human IBD pathophysiology.

Taken together, GLP-1RAs may be optimally positioned as adjunctive early-phase interventions to delay or prevent irreversible structural damage in IBD, particularly in patients with fluctuating disease activity but no established complications (17, 29). While current biologic therapies of IBD focus primarily on cytokine blockade and immune suppression (30, 31), GLP-1RAs may support mucosal healing, epithelial repair, and anti-inflammatory responses, offering a distinct therapeutic profile (8, 9). Optimizing localized delivery strategies, including nanoparticle systems and colon-targeted formulations, may further enhance their therapeutic index and support future development of combinatorial or staged therapeutic approaches (12).

In contrast, the pooled analysis for IBD-related complications demonstrated substantial uncertainty. Although the overall direction favored GLP-1RA use (RR = 0.39), the confidence interval crossed the null and statistical heterogeneity was extremely high (I^2^ = 98.9%) (16–18). Sensitivity analysis showed marked shifts in pooled estimates with the exclusion of individual studies [e.g., Adekolu et al. (17)], and meta-regression did not identify any statistically significant moderators (17). This heterogeneity likely reflects substantial differences in underlying patient populations, clinical definitions of outcomes, and data quality across studies. Importantly, it suggests that while a protective effect is plausible, its consistency is population- and context-dependent. However, due to the limited number of studies and lack of stratified outcome reporting, subgroup analyses by disease type or comorbidity were not feasible in this review.

Moreover, complication-related outcomes such as hospitalization or corticosteroid use are susceptible to contextual variation, including healthcare access, physician discretion, and insurance coverage, which may limit their reliability as indicators of biological treatment efficacy (32, 33). For instance, hospitalization thresholds may differ between countries or healthcare systems, and corticosteroid prescribing can vary according to institutional protocols (34). This reinforces the need for harmonized outcome definitions and rigorous stratification by comorbidity and disease activity in future studies.

We propose that future studies investigating complication endpoints prioritize patients with metabolic comorbidities (e.g., obesity, type 2 diabetes), who may exhibit heightened responsiveness to GLP-1RAs via immune-metabolic modulation (7, 16, 17). Given that these populations are often underrepresented in traditional IBD trials, real-world data synthesis by this review provides critical early insights (7, 16, 17). Moreover, incorporating harmonized and objective outcomes—such as mucosal healing rates, endoscopic indices, fecal calprotectin, or validated patient-reported outcomes—would reduce variability and improve interpretability (11). Exploration of long-term endpoints such as corticosteroid-free remission, surgery-free survival, or need for biologic escalation may further elucidate GLP-1RA’s role in disease trajectory modification.

In addition to limitations inherent to the evidence base, future research should explore the translational potential of combining GLP-1RAs with current therapeutic standards (35, 36). Given their favorable safety profile and complementary mechanism of action, GLP-1RAs might be used alongside biologics, particularly in cases where conventional agents fail to achieve mucosal healing or in patients with comorbid metabolic syndrome (18, 37–39). Synergistic treatment models may allow for dosage reduction of immunosuppressants and minimize long-term adverse events, an avenue deserving rigorous exploration through controlled trials.

Several limitations in the underlying evidence warrant consideration. All included studies were observational cohorts, subject to selection bias and residual confounding. Definitions of complications varied substantially, and most studies lacked key endpoints such as endoscopic healing or patient-centered outcomes. Furthermore, most studies did not stratify by IBD phenotype (Crohn’s disease vs. ulcerative colitis), disease location, or background therapies, limiting interpretability and generalizability (7, 15, 17, 18). While some included studies reported individual components of complications (e.g., hospitalization or corticosteroid use), these outcomes could not be meta-analyzed separately due to the small number of eligible studies and inconsistent definitions. Notably, 3 of the 6 included studies were abstract-only conference reports, which were retained to enable synthesis in this nascent and under-researched area. However, the inclusion of non–peer-reviewed abstracts may introduce reporting bias, limited methodological transparency, and elevated risk of heterogeneity, particularly in complication-related outcomes. This limitation was accounted for in our GRADE assessment and is transparently acknowledged in the abstract and discussion. As this research area remains in its early stages, the current analysis aimed to identify preliminary signals of association and stimulate further investigation.

We took steps to mitigate bias by following PRISMA 2020 guidelines, conducting comprehensive database searches, applying dual independent screening and extraction, and using NOS and GRADE frameworks (20, 22, 23). Nonetheless, limitations persist. The small number of eligible studies precluded detailed subgroup analysis (n = 6). One study required estimation of confidence intervals from raw data. Meta-regression was underpowered due to limited and inconsistently reported moderators. Registered but unpublished data may have been missed, and language restrictions may have led to exclusion of relevant non-English studies. Additionally, the literature search did not include databases such as SCOPUS or CINAHL, which may have limited the comprehensiveness of the review and introduced the risk of missing potentially relevant studies.

These findings have implications across clinical practice, health policy, and research. Clinically, GLP-1RAs hold promise as repurposed agents for use in metabolically high-risk IBD subgroups, supported by their safety profile and biologic plausibility (14–18, 37). As the included cohorts were predominantly composed of individuals with obesity or type 2 diabetes, the applicability of these findings is most relevant to metabolically enriched IBD subgroups. These findings should not be generalized to normometabolic IBD patients in the absence of supporting data. Strategically, they may serve as complements to traditional immunosuppressants by targeting epithelial repair and systemic inflammation. From a policy perspective, our results support the development of cross-indication reimbursement frameworks and updated treatment algorithms that integrate metabolic comorbidity management into IBD care.

Future research should prioritize mechanism-informed randomized trials and prospective cohort studies that target metabolically defined or treatment-naive IBD subgroups. These studies should explore targeted mucosal delivery strategies, such as nanoparticle-based or colonic-release formulations, and incorporate standardized, clinically meaningful endpoints including mucosal healing, endoscopic remission, and patient-reported outcomes to improve translational relevance. By advancing research across these axes, we can move closer to personalized, mechanism-aligned, and risk-stratified treatment models for IBD that incorporate GLP-1RAs into the therapeutic landscape.

## 5 Conclusion

In conclusion, this meta-analysis synthesizing six cohort studies demonstrates that GLP-1 receptor agonist use is associated with a 55% reduction in IBD-related surgery risk and a potential 61% reduction in complications. These findings support emerging mechanistic evidence for the anti-inflammatory and epithelial-regenerative roles of GLP-1 signaling. With a favorable safety profile and potential metabolic benefits, GLP-1RAs may offer additive value alongside conventional biologics, particularly in patients with comorbid obesity or T2D. Future prospective studies with harmonized, objective outcomes are warranted to define GLP-1RAs’ position in IBD care and confirm their role in altering disease progression.

## Data Availability

The original contributions presented in the study are included in the article/Supplementary material, further inquiries can be directed to the corresponding authors.
