# Supplementary material for: The role of GLP-1 receptor agonists in IBD-related surgery and IBD-related complications of inflammatory bowel disease among patients with metabolic comorbidities: a systematic review and meta-analysis
Source: Front Med (Lausanne). 2025 Aug 21;12:1621958. doi: 10.3389/fmed.2025.1621958 (PMC12408605; doi:10.3389/fmed.2025.1621958)
Supplement: Supplementary file 1 [file Data_Sheet_1.docx]

**Supplementary 1. Leave-One-Out Sensitivity Analysis –IBD-related Surgery Outcome**

This figure shows the results of a leave-one-out sensitivity analysis for IBD-related surgery. For each study listed on the y-axis, the pooled effect estimate was recalculated with that study omitted. Circles represent the new point estimates, and the horizontal lines denote the corresponding 95% confidence intervals. The relative stability across estimates indicates that no individual study had an undue influence on the overall result. Desai2024 appears twice due to distinct cohorts or endpoints reported within the same publication.

**Supplementary 2: Leave-one-out sensitivity analysis for IBD-related complications**

This figure presents the results of a leave-one-out sensitivity analysis evaluating the robustness of the meta-analysis on GLP-1 receptor agonists and IBD-related complications. For each study listed on the y-axis, the pooled effect estimate was recalculated after omitting that study. Circles indicate the revised point estimates, and horizontal lines represent 95% confidence intervals. The estimates remained directionally consistent across exclusions, suggesting overall robustness. Notably, removal of Adekolu2024 substantially reduced the estimated treatment effect and narrowed the confidence interval, indicating that this study may have contributed significantly to the observed heterogeneity in the overall analysis.
