# Supplementary material for: The role of GLP-1 receptor agonists in IBD-related surgery and IBD-related complications of inflammatory bowel disease among patients with metabolic comorbidities: a systematic review and meta-analysis
Source: Front Med (Lausanne). 2025 Aug 21;12:1621958. doi: 10.3389/fmed.2025.1621958 (PMC12408605; doi:10.3389/fmed.2025.1621958)
Supplement: Supplementary file 3 [file Table_2.docx]

**Search Strategy**

The effect of GLP-1 receptor agonists on IBD-related surgeries and complications: A meta-analysis of cohort studies.

|  | **Medline/Pub Med** | **Results** |
| --- | --- | --- |
| 1 | (GLP-1* OR GLP-1 receptor agonist* OR Glucagon-like peptide-1* OR GLP-1RA* OR Semaglutide* OR Liraglutide* OR Tirzepatide* OR Dulaglutide* OR Exenatide* OR Albiglutide* OR Lixisenatide* OR Efpeglenatide*).mp. [mp=title, book title, abstract, original title, name of substance word, subject heading word, floating sub-heading word, keyword heading word, organism supplementary concept word, protocol supplementary concept word, rare disease supplementary concept word, unique identifier, synonyms, population supplementary concept word, anatomy supplementary concept word] | 30,042 |
| 2 | (Inflammatory bowel disease* OR Crohn's disease* OR Ulcerative colitis* OR IBD pathophysiology* OR Chronic intestinal inflammation* OR IBD management* OR IBD therapies* OR Gastrointestinal inflammation*). mp. [mp=title, book title, abstract, original title, name of substance word, subject heading word, floating sub-heading word, keyword heading word, organism supplementary concept word, protocol supplementary concept word, rare disease supplementary concept word, unique identifier, synonyms, population supplementary concept word, anatomy supplementary concept word] | 137,727 |
| 3 | (Clinical trials* OR Randomized controlled trials* OR Clinical outcomes* OR Intervention studies* OR Treatment efficacy* OR Patient outcomes* OR Disease progression*).mp. [mp=title, book title, abstract, original title, name of substance word, subject heading word, floating sub-heading word, keyword heading word, organism supplementary concept word, protocol supplementary concept word, rare disease supplementary concept word, unique identifier, synonyms, population supplementary concept word, anatomy supplementary concept word] | 1,280,682 |
| 4 | (Cohort studies* OR Longitudinal studies* OR Retrospective cohort* OR Prospective cohort* OR Disease incidence* OR Follow-up studies* OR Population-based studies*).mp. [mp=title, book title, abstract, original title, name of substance word, subject heading word, floating sub-heading word, keyword heading word, organism supplementary concept word, protocol supplementary concept word, rare disease supplementary concept word, unique identifier, synonyms, population supplementary concept word, anatomy supplementary concept word] | 1,410,229 |
| 5 | (Real-world data* OR Real-world evidence* OR RWE* OR Electronic health records* OR Patient-reported outcomes* OR Pragmatic trials*).mp. [mp=title, book title, abstract, original title, name of substance word, subject heading word, floating sub-heading word, keyword heading word, organism supplementary concept word, protocol supplementary concept word, rare disease supplementary concept word, unique identifier, synonyms, population supplementary concept word, anatomy supplementary concept word] | 23,885 |
| 6 | 3 or 4 or 5 | 2,582,893 |
| 7 | 1 and 2 and 6 | 17 |
| 8 | Limit 7 to English language | 17 |

|  | **Embase** | **Results** |
| --- | --- | --- |
| 1 | (GLP-1* OR GLP-1 receptor agonist* OR Glucagon-like peptide-1* OR GLP-1RA* OR Semaglutide* OR Liraglutide* OR Tirzepatide* OR Dulaglutide* OR Exenatide* OR Albiglutide* OR Lixisenatide* OR Efpeglenatide*).mp. [mp=title, abstract, heading word, drug trade name, original title, device manufacturer, drug manufacturer, device trade name, keyword heading word, floating subheading word, candidate term word] | 62,911 |
| 2 | (Inflammatory bowel disease* OR Crohn's disease* OR Ulcerative colitis* OR IBD pathophysiology* OR Chronic intestinal inflammation* OR IBD management* OR IBD therapies* OR Gastrointestinal inflammation*).mp. [mp=title, book title, abstract, original title, name of substance word, subject heading word, floating sub-heading word, keyword heading word, organism supplementary concept word, protocol supplementary concept word, rare disease supplementary concept word, unique identifier, synonyms, population supplementary concept word, anatomy supplementary concept word] | 255,734 |
| 3 | (Clinical trials* OR Randomized controlled trials* OR Clinical outcomes* OR Intervention studies* OR Treatment efficacy* OR Patient outcomes* OR Disease progression*).mp. [mp=title, book title, abstract, original title, name of substance word, subject heading word, floating sub-heading word, keyword heading word, organism supplementary concept word, protocol supplementary concept word, rare disease supplementary concept word, unique identifier, synonyms, population supplementary concept word, anatomy supplementary concept word] | 1,260,455 |
| 4 | (Cohort studies* OR Longitudinal studies* OR Retrospective cohort* OR Prospective cohort* OR Disease incidence* OR Follow-up studies* OR Population-based studies*).mp. [mp=title, book title, abstract, original title, name of substance word, subject heading word, floating sub-heading word, keyword heading word, organism supplementary concept word, protocol supplementary concept word, rare disease supplementary concept word, unique identifier, synonyms, population supplementary concept word, anatomy supplementary concept word] | 494,297 |
| 5 | (Real-world data* OR Real-world evidence* OR RWE* OR Electronic health records* OR Patient-reported outcomes* OR Pragmatic trials*).mp. [mp=title, book title, abstract, original title, name of substance word, subject heading word, floating sub-heading word, keyword heading word, organism supplementary concept word, protocol supplementary concept word, rare disease supplementary concept word, unique identifier, synonyms, population supplementary concept word, anatomy supplementary concept word] | 45,062 |
| 6 | 3 or 4 or 5 | 1,738,472 |
| 7 | 1 and 2 and 6 | 69 |
| 8 | Limit 7 to English language | 69 |

|  | **Web of science** | **Results** |
| --- | --- | --- |
| 1 | ALL=((GLP-1 receptor agonist* OR Glucagon-like peptide-1* OR Semaglutide* OR Liraglutide* OR Tirzepatide* OR Dulaglutide* OR Exenatide* OR Albiglutide* OR Lixisenatide* OR Efpeglenatide*)) | 38,022 |
| 2 | ALL=((Inflammatory bowel disease* OR Crohn's disease* OR Ulcerative colitis* OR IBD pathophysiology* OR Chronic intestinal inflammation* OR IBD management* OR IBD therapies* OR Gastrointestinal inflammation*)) | 231,228 |
| 3 | ALL=((Clinical trials* OR Randomized controlled trials* OR Clinical outcomes* OR Intervention studies* OR Treatment efficacy* OR Patient outcomes* OR Disease progression*)) | 3,502,531 |
| 4 | ALL=((Cohort studies* OR Longitudinal studies* OR Retrospective cohort* OR Prospective cohort* OR Disease incidence* OR Follow-up studies* OR Population-based studies*)) | 1,361,144 |
| 5 | ALL=((Real-world data* OR Real-world evidence* OR RWE* OR Electronic health records* OR Patient-reported outcomes* OR Pragmatic trials*)) | 367,840 |
| 6 | 3 or 4 or 5 | 4,613,641 |
| 7 | 1 and 2 and 6 | 99 |
| 8 | Limit 7 to English language | 75 |
