# Supplementary material for: The role of GLP-1 receptor agonists in IBD-related surgery and IBD-related complications of inflammatory bowel disease among patients with metabolic comorbidities: a systematic review and meta-analysis
Source: Front Med (Lausanne). 2025 Aug 21;12:1621958. doi: 10.3389/fmed.2025.1621958 (PMC12408605; doi:10.3389/fmed.2025.1621958)
Supplement: Supplementary file 4 [file Table_3.docx]

| **Study ID** | **Selection (4)** | **Comparability (2)** | **Outcome (3)** | **Total Score** | **Risk of Bias** |
| --- | --- | --- | --- | --- | --- |
| Abbound2024 | 4 | 2 | 3 | 9 | High |
| Villumsen2021 | 4 | 2 | 2 | 8 | Moderate to High |
| Nanah2024 | 4 | 1 | 2 | 7 | Moderate |
| Saadeh2024 | 4 | 1 | 3 | 8 | Moderate to High |
| Adekolu2024 | 4 | 2 | 3 | 9 | High |
| Desai2024 | 3 | 2 | 2 | 7 | Moderate |

**Table 2. Risk of Bias Assessment Using the NOS.**

(Detailed NOS scoring for each included cohort study, covering domains of selection, comparability, and outcome. Total scores were used to categorize study quality as high, moderate to high, or moderate.)
