# Supplementary material for: The role of GLP-1 receptor agonists in IBD-related surgery and IBD-related complications of inflammatory bowel disease among patients with metabolic comorbidities: a systematic review and meta-analysis
Source: Front Med (Lausanne). 2025 Aug 21;12:1621958. doi: 10.3389/fmed.2025.1621958 (PMC12408605; doi:10.3389/fmed.2025.1621958)
Supplement: Supplementary file 5 [file Table_4.docx]

| **Outcome** | **No. of Studies** | **Study Design** | **Risk of Bias** | **Inconsistency** | **Indirectness** | **Imprecision** | **Publication Bias** | **Summary of Findings** | **Overall Certainty** |
| --- | --- | --- | --- | --- | --- | --- | --- | --- | --- |
| **IBD-related Surgery** | 4 | Observational | Not serious | Not serious | Not serious | Not serious | Not detected | GLP-1RA associated with significantly reduced risk (RR=0.45, 95% CI: 0.35–0.59); I² = 38.1% | Moderate |
| **IBD-related Complications** | 4 | Observational | Serious | Serious | Not serious | Serious | Suspected | Trend toward benefit (RR=0.39, 95% CI: 0.15–1.03) but with high heterogeneity (I² = 98.9%) | Low |

**Supplementary Table 2: GRADE summary of evidence certainty.**

Certainty ratings are shown for IBD-related surgery and complication outcomes. Surgery was graded as moderate certainty based on consistency and low risk of bias; complications were graded as low certainty due to high heterogeneity, borderline significance, and possible publication bias.
