# Supplementary material for: The role of GLP-1 receptor agonists in IBD-related surgery and IBD-related complications of inflammatory bowel disease among patients with metabolic comorbidities: a systematic review and meta-analysis
Source: Front Med (Lausanne). 2025 Aug 21;12:1621958. doi: 10.3389/fmed.2025.1621958 (PMC12408605; doi:10.3389/fmed.2025.1621958)
Supplement: Supplementary file 6 [file Table_5.docx]

**Supplementary Table 4.** **Overview of Studies Eligible for Systematic Review but Excluded from Meta-analysis, with Justification.**

| **Article Title** | **Included in Meta-analysis** | **Reason if Excluded** |
| --- | --- | --- |
| Benefits of GLP-1 Agonists Use in Patients With IBD | Yes (surgery + complication) | – |
| GLP-1 Based Therapies and Risk of IBD: Real-world Evidence | No | Reported only general IBD risk; not surgery or complication-specific |
| Impact of GLP-1 Agonists on the Severity of IBD | No | Reported only biomarker (CRP); no clinical endpoints |
| Clinical Outcomes of GLP-1 in CD/UC: Insights from Large-Scale Cohort | No | No relevant outcome reported |
| GLP-1RA Associated With Improved Outcomes in IBD | No | Reported only hospitalization/emergency visit |
| Clinical Effectiveness of GLP-1 Agonists in IBD | No | Reported only CRP outcomes |
| Outcomes of IBD on GLP-1 Agonists: PSM Analysis | Yes (complication + steroid) | – |
| Risk of IBD Complications in Obese Patients on GLP-1 Therapy | Yes (complication) | – |
| Weight Loss and Safety of GLP-1 Agonists in IBD | Yes (complication + steroid + hospitalization) | – |
| GLP-1 Agonist Use Associated With Lower Complications in IBD: National Database | Yes (complication + hospitalization) | – |
| Impact of GLP-1 on Inflammatory Biomarkers in IBD | No | Only reported CRP and calprotectin |
| GLP-1 Therapies and Disease Course in IBD | Yes (surgery) | – |
| GLP-1 Agonists and Lower IBD-Related Complications in Bariatric Surgery Patients | No | Reported hospitalization, mortality; not specific to IBD surgery/complication |
| GLP-1 Agonists in Obese Patients With IBD: Clinical + Practical Recommendations | No | Narrative review |
| GLP-1 + DPP4i for T2DM and IBD Outcomes | Yes (surgery + colectomy) | – |
| Efficacy and Safety of GLP-1 in Non-diabetic IBD Patients | No | No outcome reported |
| CV Events After GLP-1RA in Immune-mediated IBD | No | Only reported mortality |
| Effect of Semaglutide on Liver Enzymes/Markers in T2DM + Obesity | No | Only CRP reported |
| Safety and Effectiveness of GLP-1 in Obese IBD Patients | No | Case report |
| Semaglutide Use in IBD + Obesity: Weight Loss Outcome | No | Case report |
| GLP-1RA in Obese IBD Patients: Molecular to Clinical Recommendations | No | Narrative review |
| GLP-1RA + Endoscopic Sleeve + ESG in IBD Patient | No | Case report |

**Note:** This table summarizes the 22 studies that met the overall eligibility criteria for the systematic review. Of these, only 6 studies were included in the meta-analysis due to the availability of extractable effect sizes for the predefined primary outcomes: IBD-related surgery or IBD-related complications. Studies that did not report these outcomes, provided only surrogate endpoints (e.g., CRP, hospitalization, or mortality), or were narrative reviews or case reports, were excluded from the quantitative synthesis but retained in the qualitative review process. This transparent classification supports alignment with PRISMA 2020 recommendations and highlights the current limitations in the available literature on GLP-1RA use in IBD.
